# Arthroscopic Supraspinatus Tendon Reconstruction Using Fascia Lata Autograft Augmentation for Type B and C Musculotendinous Junction Tears

**DOI:** 10.1016/j.eats.2025.103895

**Published:** 2025-09-18

**Authors:** Xueyan Zhao, Xiaoli Gou, Guo Zheng, Binghua Zhou

**Affiliations:** a Department of Sports Medicine, First Affiliated Hospital of Army Medical University, Chongqing, China; b Department of Orthopedic, The 71st Group Army Hospital of the Chinese People’s Liberation Army Ground Force, Xuzhou, China

## Abstract

Type B and C musculotendinous junction tears present unique surgical challenges because of poor tissue integrity. We describe an arthroscopic technique—the supraspinatus tendon reinforcement reconstruction technique—that combines autologous fascia lata grafting with remnant tendon augmentation. This biologically optimized approach achieves anatomic restoration through 3 key principles: intramuscular fascia lata passage to enhance medial integration, lateral bridging reinforcement fixation to improve load distribution, and scapular spine anchoring for structural stability. By preserving native kinematics while augmenting repair integrity, this reproducible method may reduce retear rates in type B and C musculotendinous junction tears, offering a viable alternative to traditional reconstructions.

Rotator cuff injuries predominantly manifest at or near the bone-tendon interface. However, in rare instances, tears may occur at the myotendinous junction (MTJ), characterized by medial tendon failure either within the tendon substance or at the musculotendinous junction.^1^ Although the exact pathogenesis of medial cuff tears remains incompletely elucidated, current evidence suggests a multifactorial etiology involving both acute and chronic trauma, often in conjunction with pre-existing subacromial impingement.^2^ The relative rarity of these injuries has resulted in limited literature addressing optimal repair techniques.^3^

Millett et al.^4^ established a classification system for MTJ injuries, identifying 3 distinct tear patterns and corresponding surgical approaches. Type A lesions show adequate medial tendon length and may be managed using advanced suture bridging techniques, including double-row constructs with margin convergence sutures to approximate the lateral tendon stump to the medial muscular portion. Type B injuries present with deficient medial tendon but preserved muscle quality, whereas type C tears exhibit both tendon deficiency and muscle retraction with atrophy. For type C patterns, current treatment options include superior capsule reconstruction and latissimus dorsi tendon transfer.^4^^,^^5^ Alternative approaches have been proposed, including the dynamic convergence suture bridge technique for MTJ repairs.^3^ Various bridging techniques using patch augmentation have been described to replicate the supraspinatus tendon anatomy^6^, ^7^, ^8^; however, these methods require cautious application because of concerningly high retear rates at the graft-tendon interface.^9^

The supraspinatus tendon reconstruction (STR) technique using autogenous fascia lata (FL) grafts was developed to address irreparable posterosuperior massive rotator cuff tears.^10^ Building on this foundation, we present a reinforcement reconstruction technique that incorporates the residual supraspinatus tendon. The technique preserves the benefits of traditional STR while optimizing the biomechanical advantages of remnant tendon utilization and minimizing the retear risk for enhanced long-term outcomes. A schematic of graft placement is shown in Figure 1. A comprehensive analysis of the advantages and limitations of our technique is presented in Table 1, with key technical considerations detailed in Table 2. Our clinical research was approved by the ethics committee of the First Affiliated Hospital of Army Medical University (No. BIIT2025113).

**Fig 1:**
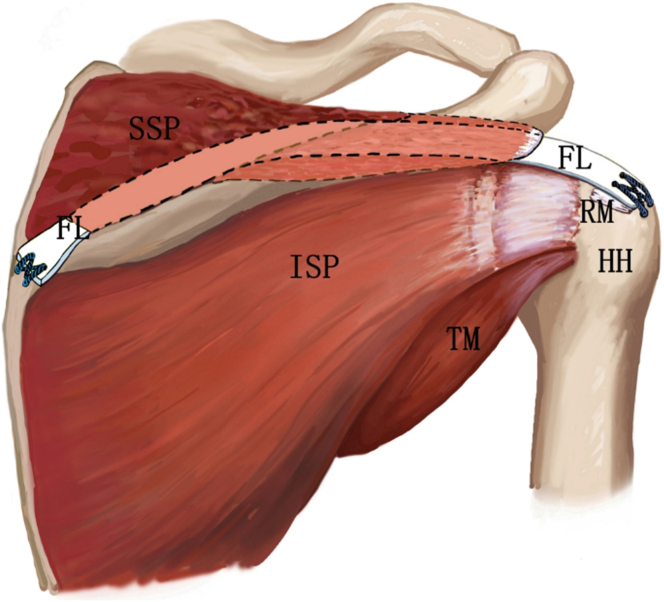
Diagram showing the surgical technique for arthroscopic supraspinatus tendon reinforcement reconstruction using autologous fascia lata (FL). The FL graft is bridged to the lateral residual stump of the supraspinatus tendon via a mattress suture, traverses the supraspinatus (SSP) muscle belly, and is finally anchored to the medial scapular spine using suture fixation to optimize fascia-muscle integration. (HH, humeral head; ISP, infraspinatus; RM, remnant; TM, teres minor.)

**Table 1:** Advantages and Disadvantages of Fascia Lata Reconstruction

Advantages
Excellent interface healing ability between fascia lata and both bone and muscle
Effective for type B and C musculotendinous junction tears
Dynamic supraspinatus reconstruction with favorable biomechanical properties
Technically reproducible with high clinical applicability
Disadvantages
Donor-site morbidity including persistent pain and scarring
Requirement for 6 wk of immobilization

**Table 2:** Pearls and Pitfalls

Pearls	Pitfalls
Harvesting the fascia lata 2 cm proximal to the greater trochanter provides optimal tissue thickness for reconstruction.	Inadequate fascia lata harvest may compromise repair integrity.
Proximal graft braiding facilitates smooth intramuscular passage during traction.	Insufficient suture reinforcement may lead to graft rupture during traction.
Immediate distal suture tightening during subacromial placement ensures complete defect coverage.	Thin scapular spine bone increases the risk of screw penetration or pullout during fixation.

## Surgical Technique

### Indications

The described technique can be used to repair type B and type C MTJ injuries. A schematic of the arthroscopic supraspinatus tendon reinforcement reconstruction technique is shown in Figure 1.

### Patient Evaluation, Imaging, and Indications

**Fig 2:**
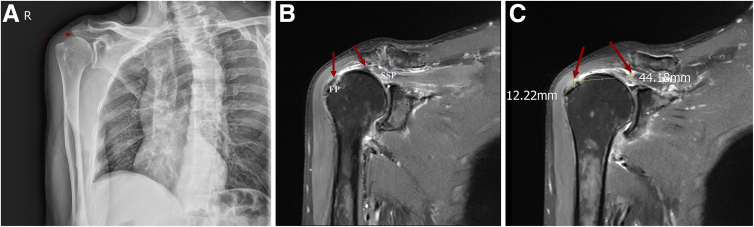
(A) Radiographic examination of the right shoulder joint in the anteroposterior view shows narrowing of the subacromial space, suggesting subacromial impingement syndrome. (R, right.) (B, C) Coronal magnetic resonance imaging of the right shoulder joint shows the residual tissue of the torn supraspinatus tendon (SSP) on the right side (red arrows). The images reveal a massive SSP tear, with the distal tendon stump being retracted medially and of poor tissue quality. (FP, footprint.)

A thorough preoperative evaluation is paramount for patients with suspected MTJ injuries because clinical and imaging findings directly inform surgical decision making. The diagnostic triad of shoulder pain, functional impairment, and weakness, when correlated with characteristic magnetic resonance imaging (MRI) findings, establishes the diagnosis of MTJ pathology.^11^ Initial radiographic evaluation provides valuable preliminary assessment of the osseous architecture, including quantification of subacromial space narrowing and identification of osteophyte formation (Fig 2A). However, MRI emerges as the diagnostic modality of choice for MTJ injuries because of its unparalleled soft-tissue resolution, enabling comprehensive evaluation of residual tendon quality at both the proximal and distal segments, precise characterization of tear morphology and retraction patterns, and assessment of concomitant muscle degeneration (Fig 2B).

After confirmation of a type B or C MTJ injury through this multimodal diagnostic approach, patients may be counseled regarding arthroscopic examination and potential reconstructive options. This preoperative phase should include detailed discussion of surgical objectives, anticipated outcomes, and rehabilitation requirements to facilitate informed decision making.

### Anesthesia and Patient Positioning

**Fig 3:**
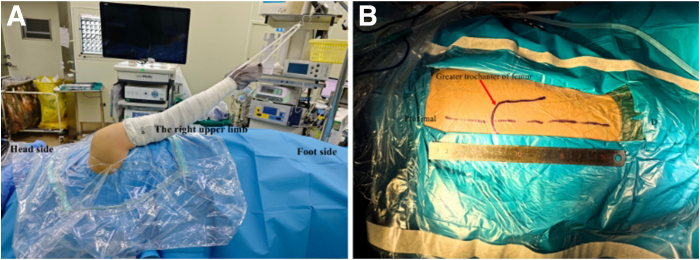
(A) Right shoulder, Left Lateral Decubitus Position. (B) Disinfection area in ipsilateral hip for harvesting of fascia lata.

After induction of general anesthesia, the patient is positioned in the lateral decubitus position with the unaffected side down (Fig 3A, Video 1). After standard surgical disinfection, sterile draping is applied. The surgical field extends from the affected upper limb and shoulder to the neck region, posteriorly to the midline of the back, anteriorly to the nipple line, and inferiorly to the ipsilateral hip and lateral thigh. A surgical incision is marked 2 cm proximal to the greater trochanter along the femoral shaft axis (Fig 3B). A sterile traction sleeve is applied to the affected limb and connected to the traction frame, maintaining 30° of abduction and 20° of forward flexion with 2 kg of traction.

### Diagnostic Arthroscopy and Subacromial Decompression

**Fig 4:**
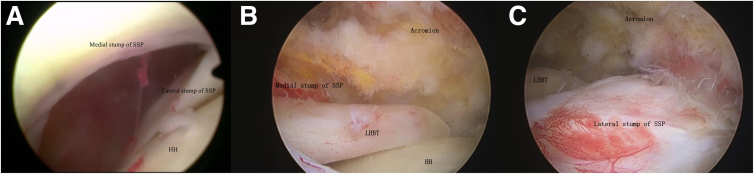
(A) Arthroscopic view of glenohumeral joint showing complete supraspinatus (SSP) tendon rupture with residual tissue in lateral footprint region (posterior portal). (B) Subacromial inspection reveals a musculotendinous junction tear of the SSP with severely degenerated residual medial tendon tissue of poor quality. (C) The residual tissue in the lateral footprint region is abundant and of good quality. (HH, humeral head; LHBT, long head of biceps tendon.)

Shoulder arthroscopy is performed using a 30° arthroscope. Initial visualization is achieved through a standard posterior portal to assess the glenohumeral joint, including evaluation of the subscapularis tendon, glenoid labrum, articular cartilage, and long head of the biceps tendon (Fig 4A). When indicated, an anterolateral working portal is established for repair procedures. In cases not requiring intra-articular repair, the arthroscope is redirected into the subacromial space through the same portal. A lateral portal is then created to perform subacromial bursectomy, decompression, and complete mobilization of the retracted rotator cuff edges. Rotator cuff tears are classified as massive type B or C MTJ lesions, precluding direct primary repair because of their complex nature (Fig 4 B and C).

### FL Harvest

**Fig 5:**
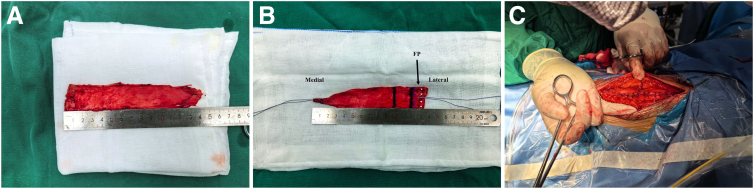
(A) Harvested fascia lata graft. (B) Prepared graft configuration with muscle-penetrating portion (inner segment) and footprint-covering portion (outer segment). The blue and white markers indicate suture anchor positions for footprint fixation. (C) Donor-site closure with side-to-side fascial approximation. (FP, footprint.)

The dimensions of the FL graft are determined by measuring the anterior-posterior width of the MTJ defect and the length from the lateral acromion to the medial scapular spine. A longitudinal incision is made along the lateral thigh of the affected limb, beginning 2 cm proximal to the greater trochanter and extending distally according to the required graft length. The FL is exposed and harvested, with careful removal of excess muscular and adipose tissue (Fig 5A). The graft is then tubularized using running sutures, braided for reinforcement, and marked for orientation (Fig 5B). The donor site is closed in layers with particular attention to achieving meticulous hemostasis to prevent hematoma formation. The residual fascial edges are approximated under tension to minimize the risk of muscle herniation (Fig 5C).

### Graft Implantation

**Fig 6:**
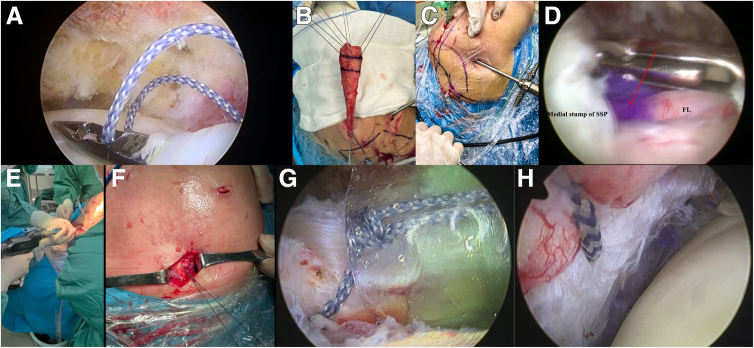
Surgical technique for fascia lata (FL) graft reconstruction. (A) Three high-strength sutures are placed into the lateral stump of the supraspinatus tendon. (B) The high-strength sutures are passed through the humeral side of the fascia lata graft ex vivo. (C) Graft passage into the subacromial space is performed under traction while under arthroscopic surveillance. (D) Proper graft positioning is confirmed when the 4-cm marking line (red arrow) aligns with the supraspinatus tendon (SSP) stump. (E) Medial fixation is performed with an absorbable anchor placed at the scapular margin. (F) Secure medial graft fixation is achieved. (G) The residual rotator cuff tissue undergoes side-to-side suturing to the graft. (H) The graft adequately covers the humeral head and is securely integrated with the residual rotator cuff tissue. (FL, fascia lata.)

The lateral stump of the supraspinatus tendon is preserved. Three high-strength sutures are placed in vertical mattress configuration through the residual tendon from anterior to posterior with equidistant spacing (Fig 6A). These sutures are retrieved through the working portal and sequentially secured to the FL graft 2 cm distal to the marking line (without knot tying at this stage) (Fig 6B). The medial scapular spine is palpated externally, followed by a 3-cm incision along its axis. Through this approach, a Kelly clamp is introduced into the supraspinatus muscle belly. Under arthroscopic visualization, the clamp tip is advanced beneath the supraspinatus stump to retrieve the graft’s traction sutures. Continuous traction is applied to deliver the FL graft into the subacromial space (Fig 6C). Traction is discontinued when the graft’s 4-cm marking line aligns with the supraspinatus tendon stump (Fig 6D). Adequate graft coverage over the tear site is confirmed. The pre-placed high-strength sutures are tensioned, secured with knots, and reinforced using a lateral-row anchor to offload tension between the graft and supraspinatus stump. Via the medial scapular incision, the graft’s medial end and underlying scapular cortex are exposed. An absorbable anchor is inserted at the medial-most aspect of the scapular spine (Fig 6E). The graft’s medial end is tensioned appropriately and fixed (Fig 6F). Finally, arthroscopic visualization is re-established in the subacromial space. Residual rotator cuff tissue is approximated to the graft with side-to-side sutures (Fig 6G). The glenohumeral joint is then entered to confirm: (1) complete graft coverage over the humeral head and (2) intimate apposition between residual cuff tissue and graft (Fig 6H). Instrumentation is removed, and the skin incisions are closed, concluding the procedure.

### Rehabilitation Protocol

During the first 6 postoperative weeks, strict shoulder immobilization must be maintained using an abduction brace. The brace may be temporarily removed for hygienic care while providing manual support to the affected limb. Patients should perform daily hand-grip exercises using a dynamometer, while maintaining full active range of motion in the wrist and elbow joints.

**Fig 7:**
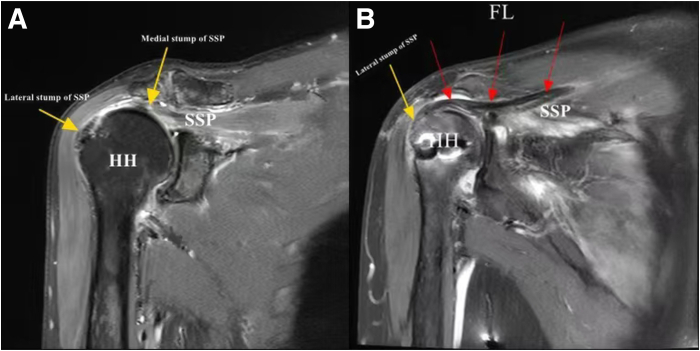
Magnetic resonance imaging findings. (A) Preoperative coronal T2-weighted fat-suppressed image showing musculotendinous junction injury with partial preservation of distal tendon tissue (yellow arrows). (B) Six-month postoperative coronal T2-weighted fat-suppressed image showing successful integration of both the preserved distal tendon (yellow arrow) and graft with the supraspinatus muscle belly (red arrows). (FL, fascia lata; HH, humeral head; SSP, supraspinatus.)

At the 6-week follow-up, supervised passive range-of-motion exercises should be initiated, progressing gradually to active-assisted and, eventually, active exercises. By 3 months postoperatively, patients may begin structured strengthening exercises. On follow-up MRI at 6 months, excellent graft integration is observed, with the FL graft showing complete incorporation with the residual supraspinatus tendon (Fig 7), representing significant improvement compared with preoperative imaging findings.

## Discussion

Although tears of the rotator cuff close to the MTJ are uncommon, the occurrence of subsequent retears has risen significantly with the growing frequency of rotator cuff repair procedures. In repairs of MTJ tears, challenges faced include the achievement of a balanced biomechanical repair construct with minimal tension and restoration of optimal tendon length; risk of suture cut-through in degenerated medial muscle fibers; and lack of sufficient medial tendon to provide an adequate working length, possibly due to retraction.^3^ Repairing the damage in this context proves challenging, and there is a scarcity of comprehensive studies focused on repair methods and their results.

Given the low incidence of these tears, there is a paucity of literature on their repair techniques and functional outcomes after repair. In 2017, Millett et al.^4^ described 3 major tear patterns of MTJ injuries and the possible repair techniques for each tear pattern. Superior capsule reconstruction, latissimus dorsi tendon transfer, and reverse total shoulder arthroplasty have been used for the treatment of type B and C MTJ tears. The STR technique presented a stepwise approach to accomplish the dual goals of stable anatomic reconstruction and restoration of the dynamic function of the supraspinatus tendon.^10^ The supraspinatus tendon reinforcement reconstruction technique involves a method similar to the STR technique using an autologous FL graft passed through the residual muscle belly. However, the FL graft in supraspinatus tendon reinforcement reconstruction achieves interface healing with the residual supraspinatus tendon, and the FL graft directly heals to the bone at the footprint in the STR technique. Previous studies have shown that the FL graft achieves excellent interface healing with the remnant tendon.^12^ In brief, the supraspinatus tendon reinforcement reconstruction technique achieves 3 key advantages: (1) enhanced early fixation strength through acromial attachment, (2) increased muscle-graft contact area promoting biological incorporation, and (3) eventual formation of a neotendon structure that restores nearly anatomic dynamics.

The use of graft augmentation in rotator cuff repairs has been growing in popularity because of its ability to enhance time-zero biomechanical repair strength and decrease retear rates.^13^^,^^14^ There are many types of grafts to choose from, such as allogeneic dermal grafts, xenogeneic dermal grafts, autologous FL grafts, and synthetic grafts. The use of xenograft in rotator cuff augmentation has yielded conflicting results. Several studies have reported a high incidence of associated complications.^15^^,^^16^ In addition, 20% of patients were noted to have hypersensitivity reactions related to the graft itself.^17^ Our technique specifically uses autologous FL for dynamic reconstruction. This approach offers several distinct advantages. Autologous tissue eliminates rejection risks and hypersensitivity reactions associated with xenografts, which have been reported in up to 20% of cases. Unlike static reconstructions, the FL’s biological properties may allow for more physiological force transmission, potential tissue remodeling, and even reversal of induced intramuscular fat infiltration.^18^ The robust nature of FL provides immediate mechanical reinforcement while serving as a scaffold for host tissue integration.

The efficacy of rotator cuff repair critically depends on successful graft integration at both the musculotendinous and bone-tendon interfaces. Although arthroscopic patch grafting with mattress suture fixation represents a technical advancement, postoperative retear rates remain concerning, ranging from 13% to 94%.^19^, ^20^, ^21^ Central to this challenge is the healing capacity at the medial muscle-graft junction. The resultant fascia-muscle complex not only restores superior glenohumeral stability but also significantly reduces medial-junction failure risk. Equally crucial is the lateral footprint interface, where FL autografts show superior biological healing and fixation strength compared with synthetic alternatives.^22^ The STR technique achieves bone-tendon integration comparable to superior capsule reconstruction while avoiding the complications associated with salvage procedures such as reverse total shoulder arthroplasty.^23^^,^^24^ This dual-interface optimization—combining robust musculotendinous incorporation with reliable bone attachment—positions STR as a physiologically sound alternative that harnesses the patient’s native healing capacity at both critical junctions. As such, this dynamic reconstruction technique represents an evolution in MTJ repair philosophy, moving beyond simple gap bridging to potentially restoring more normal force coupling across the shoulder joint. In conclusion, arthroscopic supraspinatus tendon reinforcement reconstruction represents a unique surgical option and may reduce retear rates while preserving native kinematics, offering a reproducible solution for type B and C MTJ tears.

## Disclosures

All authors (X.Z., X.G., G.Z., B.Z.) declare that they have no known competing financial interests or personal relationships that could have appeared to influence the work reported in this paper.

## Supplementary Data

Video 1 The patient is placed under general anesthesia in the contralateral decubitus position. First, the arthroscope is placed into the glenohumeral joint through the posterior portal, the lesion is evaluated and repaired through the anteral portal. Then, the arthroscope is moved to the subacromial space, and debridement and subacromial decompression are performed via the lateral portal. When a type B or C musculotendinous junction (MTJ) lesion is confirmed, the width and length of the fascia lata patch are measured. Then, the assistants begin to harvest the fascia lata according the measurements. The graft is tubularized using running sutures, braided for reinforcement. At the same time, 3 high-strength sutures are placed in a vertical mattress fashion through the tendon remnant using a bird-beak device. These sutures are retrieved through the working portal and secure the fascia lata graft 2 cm distal to the lateral ending. Polydioxanone (PDS) suture is passed along the supraspinatus muscle to the subacromial space using a clamp. The PDS suture was pulled and tracted the running sutures in the fascia lata medially until out of the skin. Then, the pre-placed high-strength sutures in the lateral fascia lata are secured with knots, using No. 2 Versalok (DePuy Mitek Synthes). In addition, an absorbable anchor (Lupine, DePuy Mitek Synthes) is inserted at the medial-most aspect of the scapular spine. The graft’s medial ending is tensioned and fixed. If the infraspinatus tendon is qualified for suturing, 2 or 3 sutures are placed between the posterior edge of the fascia lata and the infraspinatus tendon. A final inspection is performed from the subacromial space and the glenohumeral joint, respectively.

## References

[1] Lädermann A., Christophe F.K., Denard P.J., Walch G. (2012). Supraspinatus rupture at the musclotendinous junction: An uncommonly recognized phenomenon. J Shoulder Elbow Surg.

[2] Benazzo F., Marullo M., Pietrobono L. (2014). Supraspinatus rupture at the musculotendinous junction in a young woman. J Orthop Traumatol.

[3] Gatot C., Lie H.M.E., Tijauw Tjoen D.L. (2023). Arthroscopy technique: Repair of musculotendinous junction rotator cuff tears in the shoulder using a dynamic convergence suture bridge technique. Arthrosc Tech.

[4] Millett P.J., Hussain Z.B., Fritz E.M., Warth R.J., Katthagen J.C., Pogorzelski J. (2017). Rotator cuff tears at the musculotendinous junction: Classification and surgical options for repair and reconstruction. Arthrosc Tech.

[5] Hall T., Danielson K., Brandenburg S., Matelic T. (2020). A case series of recurrent myotendinous rotator cuff tears repaired and augmented with dermal allograft: Clinical outcomes at two years. J Shoulder Elbow Surg.

[6] Bi M., Zhou K., Gan K. (2021). Combining fascia lata autograft bridging repair with artificial ligament internal brace reinforcement: A novel healing-improvement technique for irreparable massive rotator cuff tears. Bone Joint J.

[7] Rhee S.M., Oh J.H. (2017). Bridging graft in irreparable massive rotator cuff tears: Autogenic biceps graft versus allogenic dermal patch graft. Clin Orthop Surg.

[8] Ono Y., LeBlanc J., Bois A.J. (2022). Graft healing is more important than graft technique: Superior capsular reconstruction versus bridging grafts—A prospective randomized controlled trial. Arthroscopy.

[9] Jones C.R., Snyder S.J. (2015). Massive irreparable rotator cuff tears: A solution that bridges the gap. Sports Med Arthrosc Rev.

[10] Ma L., Liao Y.T., Wang Z.Y., Li H.S., Tang K.L., Zhou B.H. (2023). Supraspinatus tendon reconstruction using fascia lata autograft for irreparable posterosuperior massive rotator cuff tears. Arthrosc Tech.

[11] Taneja A.K., Kattapuram S.V., Chang C.Y., Simeone F.J., Bredella M.A., Torriani M. (2014). MRI findings of rotator cuff myotendinous junction injury. AJR Am J Roentgenol.

[12] de Rezende Pinna B., Stavale J.N., de Lima Pontes P.A., de Oliveira Camponês do Brasil O. (2011). Histological analysis of autologous fascia graft implantation into the rabbit voice muscle. Braz J Otorhinolaryngol.

[13] Barber F.A., Burns J.P., Deutsch A., Labbé M.R., Litchfield R.B. (2012). A prospective, randomized evaluation of acellular human dermal matrix augmentation for arthroscopic rotator cuff repair. Arthroscopy.

[14] Bailey J.R., Kim C., Alentorn-Geli E. (2019). Rotator cuff matrix augmentation and interposition: A systematic review and meta-analysis. Am J Sports Med.

[15] D’Ambrosi R., Ragone V., Comaschi G., Usuelli F.G., Ursino N. (2019). Retears and complication rates after arthroscopic rotator cuff repair with scaffolds: a systematic review. Cell Tissue Bank.

[16] Haft M., Li S.S., Pearson Z.C., Ahiarakwe U., Bettencourt A.F., Srikumaran U. (2025). No Short-term Clinical Benefit to Bovine Collagen Implant Augmentation in Primary Rotator Cuff Repair: A Matched Retrospective Study. Clin Orthop.

[17] Jackson G.R., Bedi A., Denard P.J. (2022). Graft augmentation of repairable rotator cuff tears: An algorithmic approach based on healing rates. Arthroscopy.

[18] Liao Y., Zhou Z., Wang J., Li H., Zhou B. (2024). Fascia lata autografts achieve interface healing with the supraspinatus muscle histologically and mechanically in a rat supraspinatus tendon reconstruction model for massive irreparable rotator cuff tears. Arthroscopy.

[19] Jeong H.Y., Kim H.J., Jeon Y.S., Rhee Y.G. (2018). Factors predictive of healing in large rotator cuff tears: Is it possible to predict retear preoperatively?. Am J Sports Med.

[20] Mandaleson A. (2021). Re-tears after rotator cuff repair: Current concepts review. J Clin Orthop Trauma.

[21] Mori D., Kizaki K., Funakoshi N. (2021). Irreparable large to massive rotator cuff tears with low-grade fatty degeneration of the infraspinatus tendon: Minimum 7-year follow-up of fascia autograft patch procedure and partial repair. Am J Sports Med.

[22] Li H.S., Zhou M., Huang P. (2022). Histologic and biomechanical evaluation of the thoracolumbar fascia graft for massive rotator cuff tears in a rat model. J Shoulder Elbow Surg.

[23] Ernstbrunner L., Suter A., Catanzaro S., Rahm S., Gerber C. (2017). Reverse total shoulder arthroplasty for massive, irreparable rotator cuff tears before the age of 60 years: Long-term results. J Bone Joint Surg Am.

[24] Frankle M., Levy J.C., Pupello D. (2006). The reverse shoulder prosthesis for glenohumeral arthritis associated with severe rotator cuff deficiency. A minimum two-year follow-up study of sixty patients surgical technique. J Bone Joint Surg Am.

